# Simultaneous Cesarean Delivery and Pelvic Stabilization for Open-Book Pelvic Injury in Late Pregnancy: A Case Report

**DOI:** 10.7759/cureus.114194

**Published:** 2026-08-09

**Authors:** Wei Jie Tee, Wang Chen Kang, Chuah Jia Peng

**Affiliations:** 1 Orthopedic Surgery, Changi General Hospital, Singapore, SGP; 2 Orthopedic Surgery, Universiti Sultan Zainal Abidin, Kuala Terengganu, MYS; 3 Orthopedics and Trauma, Hospital Sultanah Aminah, Johor Bahru, MYS

**Keywords:** apc ii, closed pelvic fracture, external fixator as definitive fixation, pelvic fracture, trauma in pregnanacy

## Abstract

Trauma during pregnancy poses a dual threat to the mother and fetus, and disruption of the pelvic ring is among the most challenging injuries a trauma team can encounter in this population. Altered maternal physiology, the presence of a gravid uterus, and concerns regarding fetal radiation exposure all complicate assessment and surgical planning. Evidence to guide management is limited, leaving the sequencing of delivery and skeletal stabilization largely dependent on case-by-case clinical judgment.

A 26-year-old woman (gravida 3, para 2) at 35 weeks and 5 days of gestation sustained these injuries after being struck by a car while riding a motorcycle. She presented alert, with stable vital signs and no tachycardia (blood pressure, 127/77 mmHg; heart rate, 72 bpm; SpO₂, 98%). Focused assessment with sonography for trauma (FAST) was negative on two occasions, and cardiotocography was reassuring, with only mild uterine activity.

Following multidisciplinary discussion, an emergency lower-segment cesarean section was performed with the pelvic binder in situ, followed by pelvic external fixation using two 5-mm supra-acetabular Schanz pins and retrograde nailing of the right femur during a single three-hour operative session. A 1.9-kg girl was delivered, with Apgar scores of 4, 7, and 10 at 1, 5, and 10 minutes, respectively. Estimated blood loss was 800 mL, and two units of packed red blood cells were transfused. Pelvic reduction was maintained at two weeks, and both the mother and neonate were discharged well on day 8.

In near-term pregnancy complicated by combined pelvic and orthopedic trauma, single-session delivery and skeletal stabilization is feasible. An external fixator can provide definitive fixation for an open-book pelvic injury.

## Introduction

Trauma complicates approximately 7% to 8% of all pregnancies and is the leading non-obstetric cause of maternal death [1,2]. Maternal mortality following major trauma approaches 10%, with the highest incidence occurring during the third trimester [1]. Pelvic ring fractures are among the most serious injuries sustained in this setting. A systematic review of 33 pregnant patients with pelvic ring fractures reported maternal and fetal mortality rates of 9.1% and 42.4%, respectively, with motor vehicle collisions accounting for 63% of injuries [3]. Anterior-posterior compression type II (APC-II) injuries comprised 21.2% of the cohort and were associated with a fetal mortality rate of 42.9% [3]. Earlier series reported a comparable fetal mortality rate of approximately 35% [4]. Concomitant fractures are common, occurring in 48.5% of reported cases, and femoral shaft fractures, in particular, have been associated with adverse maternal outcomes [3].

We present a case of an APC-II pelvic fracture with a concomitant open femoral fracture in a woman at 35 weeks and 5 days of gestation, managed with simultaneous emergency cesarean section and pelvic stabilization. This report highlights the multidisciplinary decision-making process and surgical considerations involved in managing this challenging clinical scenario.

This case report was previously presented as a poster titled “Open Book Pelvic Fracture in a Pregnant Woman: How We Approach a Disaster” at the Malaysian Orthopaedic Association Annual Scientific Meeting 2023.

## Case presentation

A 26-year-old woman, gravida 3 para 2, at 35 weeks and 5 days of gestation, was involved in a road traffic accident. She was riding a motorcycle when she was struck directly on her right side by a car. She presented with pain over the right thigh and anterior pelvic region, and fetal movements were still perceived.

On arrival at the emergency department, she was alert, with a Glasgow Coma Scale score of 15/15. Her hemodynamic parameters were stable, with a blood pressure of 127/77 mmHg, heart rate of 72 bpm, and SpO₂ of 98%. She was assessed simultaneously by the trauma and obstetric teams and resuscitated according to the Advanced Trauma Life Support (ATLS) protocol. Focused assessment with sonography in trauma (FAST) was performed twice, with no evidence of intra-abdominal injury. The fetus was viable. Cardiotocography (CTG) demonstrated a normal fetal heart rate pattern, with mild uterine activity (2-3 contractions in 10 minutes).

**Figure 1:**
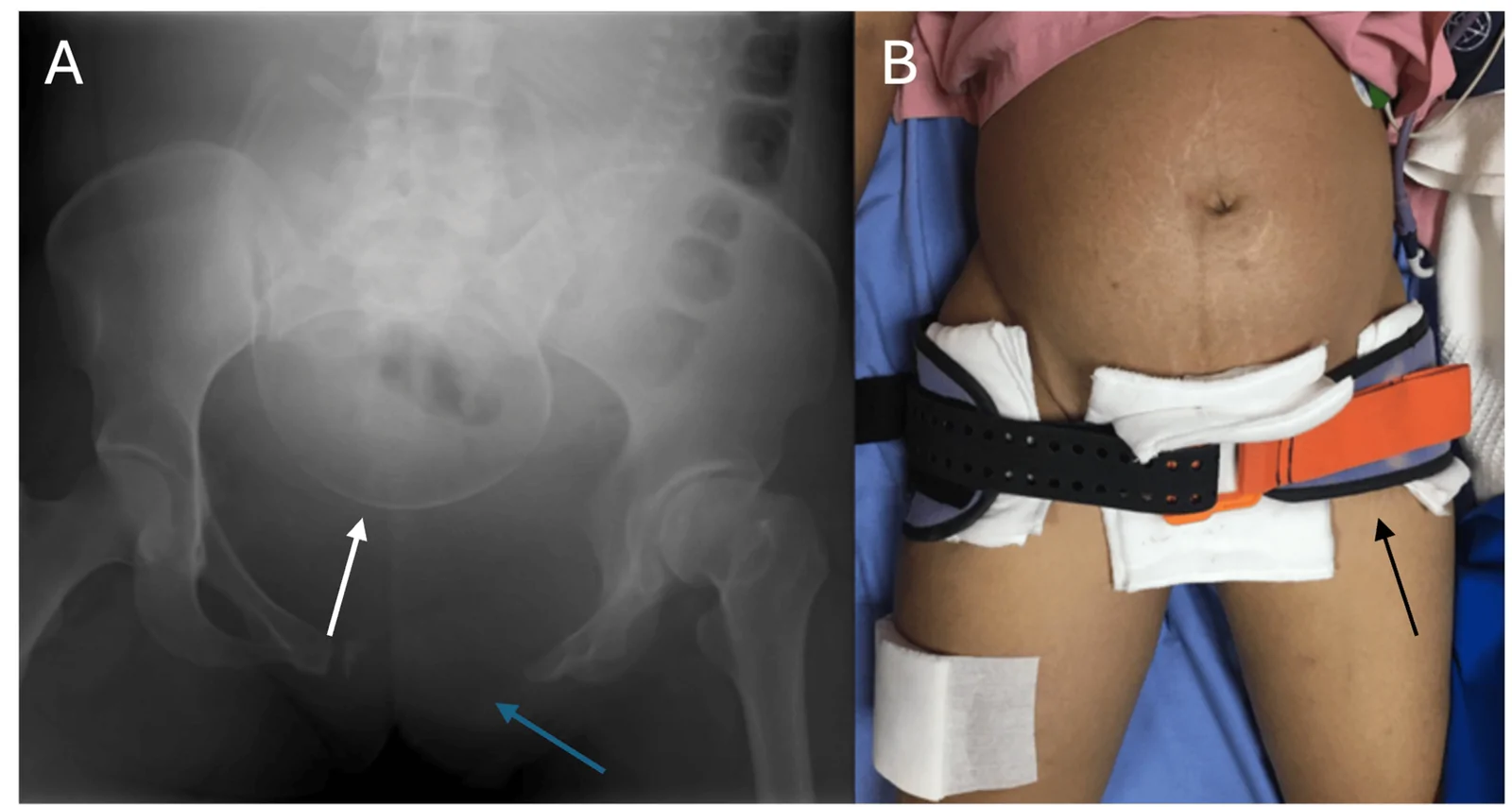
Pelvic radiograph showing open-book pelvic injury and pelvic binder application (A) Anteroposterior pelvic radiograph demonstrating an open-book pelvic injury (Young-Burgess anterior-posterior compression type II (APC-II)) (blue arrow), with the fetus in vertex position (white arrow). (B) Pelvic binder (black arrow) applied for provisional stabilization.

The secondary survey revealed tenderness over the pubic symphysis and a puncture wound over the lateral aspect of the right thigh without active bleeding, suggesting an open fracture. Radiographs obtained after informed consent demonstrated an APC-II open-book pelvic injury with pubic symphysis diastasis and an open midshaft right femoral fracture (Gustilo-Anderson type I) (Figure 1). A pelvic binder was applied immediately for provisional stabilization.

The multidisciplinary team, comprising orthopedic surgeons, obstetricians, anesthetists, and neonatologists, convened to discuss the management plan. Given the near-term gestational age (35 + 5 weeks), stable maternal and fetal parameters, pelvic instability with a risk of hemorrhage, and the need for operative access, the decision was made to proceed with coordinated emergency delivery followed by orthopedic fixation during a single operative session. Key considerations included the following: (1) the fetus was near term, with minimal benefit from delaying delivery beyond 34 weeks; (2) pelvic instability posed an ongoing risk of hemorrhage; (3) the gravid uterus would complicate positioning for fixation; and (4) a single anesthetic event was preferable to staged procedures.

**Figure 2:**
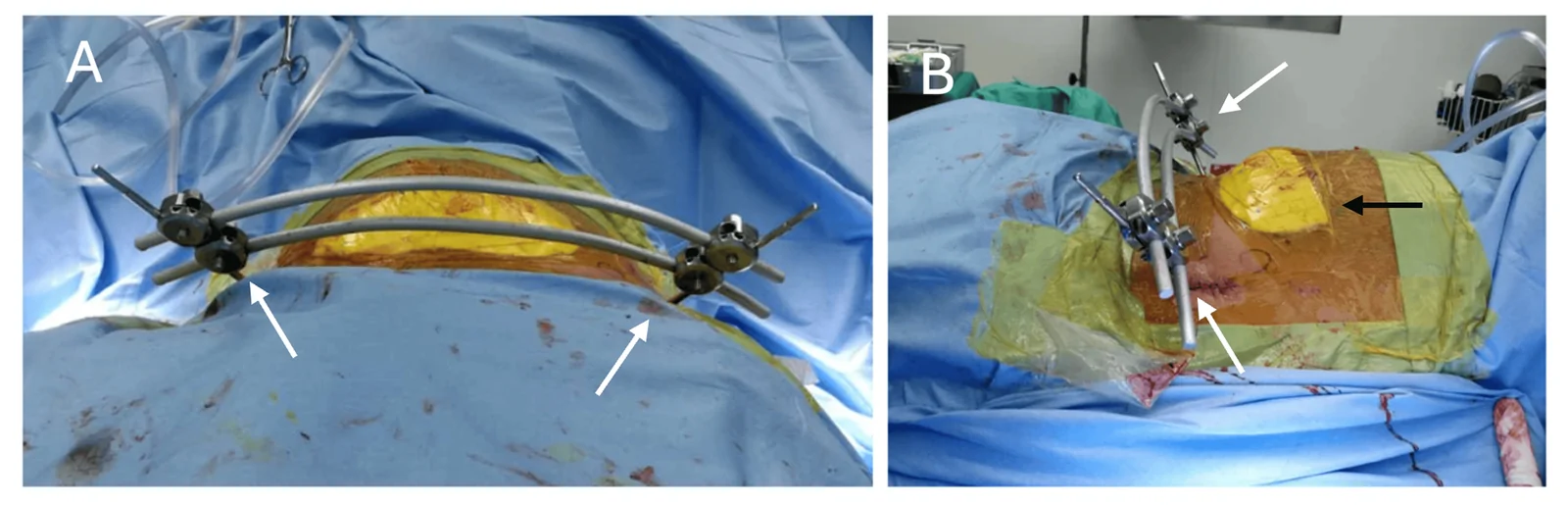
Intraoperative photographs of the pelvic external fixator with two 5-mm supra-acetabular Schanz pins (A) Anteroinferior view. (B) Lateral view. White arrows: supra-acetabular pin. Black arrow: dressed Pfannenstiel wound.

Emergency lower-segment cesarean section (EMLSCS) was performed with the pelvic binder in situ, followed by external fixation of the pelvis using two 5-mm supra-acetabular Schanz pins (Figure 2), wound debridement of the right femur, and retrograde femoral nailing. The patient delivered a baby girl weighing 1.9 kg, with Apgar scores of 4, 7, and 10 at 1, 5, and 10 minutes, respectively. The total surgical time was three hours (one hour for the obstetric procedure and two hours for the orthopedic procedures), with an estimated blood loss of 800 mL. The patient remained hemodynamically stable throughout the procedure.

**Figure 3:**
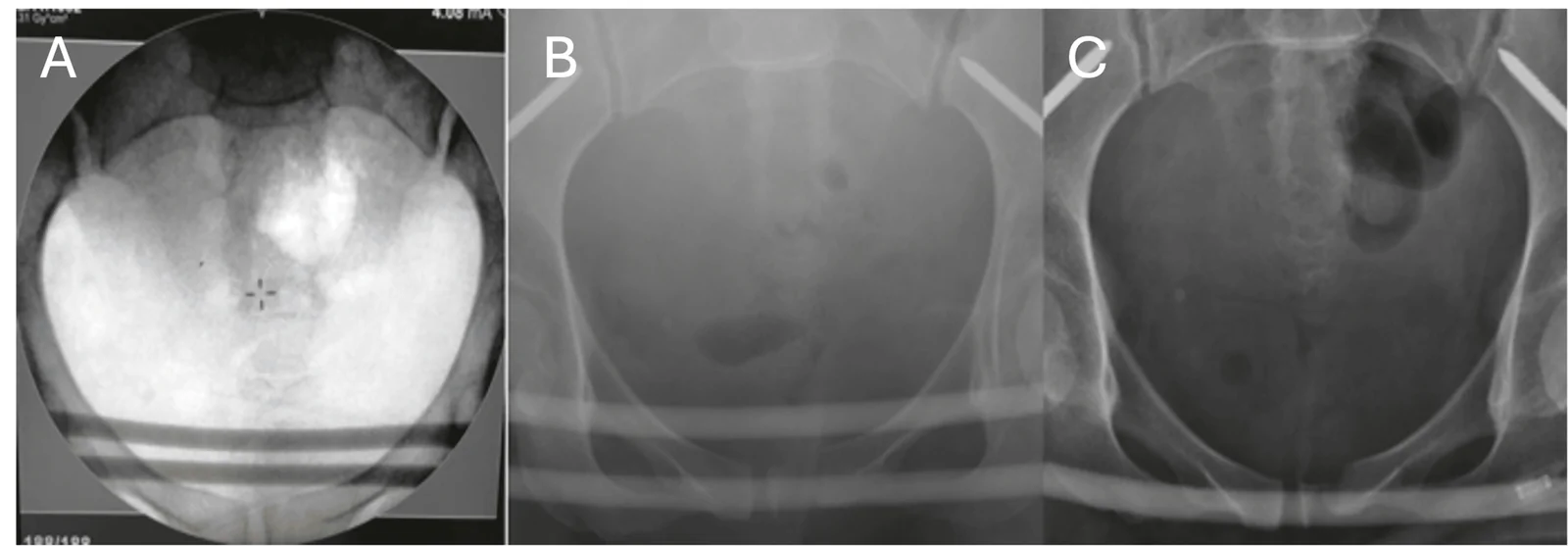
Sequential pelvic inlet radiographs showing reduction over time (A) Intraoperative reduction. (B) Immediate postoperative radiograph. (C) Two-week follow-up, showing maintained pelvic reduction with the external fixator in situ.

Postoperatively, she required transfusion of two units of packed red blood cells following a drop in hemoglobin. She was discharged on postoperative day 8, mobilizing in a wheelchair with the external fixator maintained as definitive fixation for her pelvic injury. At two-week follow-up, pelvic reduction was maintained with the external fixator in situ (Figure 3). The neonate was discharged in good condition.

Informed consent was obtained from the patient for publication of this case report and any accompanying clinical images.

## Discussion

Pelvic fractures during pregnancy present unique challenges due to the physiological changes of pregnancy, fetal vulnerability, and the need for multidisciplinary coordination.

Maternal resuscitation takes precedence, as optimal maternal stabilization offers the best chance of fetal survival [3,5]. Pregnancy increases blood volume by approximately 40%, allowing up to 1,500 mL of occult hemorrhage before signs of shock become apparent [6]. Therefore, initial hemodynamic stability does not exclude significant pelvic bleeding, and a pelvic binder was applied immediately. Aortocaval compression can reduce cardiac output by up to 30%, and a left lateral tilt of 15-30° is recommended when feasible [3,6]. Appropriate imaging should not be delayed: fetal exposure below 5 rad is not associated with an increased risk of fetal anomalies or pregnancy loss [7], and standard trauma-series radiographs deliver only a small fraction of this threshold to the uterus [7]. Abdominal shielding may further reduce exposure but can limit visualization of the fracture.

At 35 + 5 weeks, the fetus was effectively near term, with limited benefit from further gestation and no indication for antenatal corticosteroids [1], while deferring fixation would have prolonged pelvic instability and the associated hemorrhagic risk. Expedited delivery was therefore considered the most appropriate approach to protect the mother without compromising fetal outcome.

External fixation was chosen as definitive treatment for the APC-II injury, offering shorter operative time, reduced blood loss, less surgical dissection, and lower radiation exposure than internal fixation [8]. This is consistent with the report by Stohlner et al. of successful definitive external fixation for an APC-II injury during pregnancy [9]. External fixation can be applied rapidly without requiring exposure of the anterior pelvic ring, and contemporary reviews continue to favor it as the preferred initial technique during pregnancy for this reason [10]. Where the posterior ring is intact, as in this case, the frame may also serve as definitive treatment [9,11]. Supra-acetabular pins, as used in this case, provide greater purchase than iliac crest pins and keep the frame clear of the Pfannenstiel incision. Reduction quality should be assessed using validated criteria rather than subjective visual assessment. Lefaivre et al. demonstrated poor interobserver reliability for visual assessment (κ = 0.306) and validated an absolute displacement method, with displacement of >10 mm generally considered significant [12]. By this standard, reduction was maintained throughout follow-up in our patient.

Operative management of pelvic fractures during pregnancy has been reported only in isolated cases. The largest synthesis to date included only 33 patients over 23 years, with no significant difference in mortality between surgical and conservative management. Eight patients were managed with an external fixator, which was always applied before delivery [3]. Our case differs in that the external fixator was applied immediately after delivery during the same anesthetic. Individual comparators include Stohlner et al. (definitive external fixation for an APC-II injury) [9], Pisoudeh et al. (combined pelvic ring and sacral fracture) [13], Fleifel et al. (subcutaneous internal fixation for symphyseal diastasis) [14], Tomer et al. (conservative management of hip dislocation) [15], and Almog et al. (combined conservative and operative approaches) [16]. Amorosa et al. recommend applying the same surgical indications used for non-pregnant patients and note that symphyseal fixation can be performed through the same Pfannenstiel incision used for cesarean delivery when the fetus is near term [1]. In our case, external fixation was selected because of its lower soft-tissue insult.

This combined approach was feasible because delivery first removed the gravid uterus as an obstacle to positioning, reduction maneuvers, and fluoroscopy, while also eliminating concerns regarding fetal radiation exposure. A single anesthetic avoided a second induction and shortened the period of pelvic instability. The procedures were completed within three hours (one hour for the obstetric procedure and two hours for the orthopedic procedures), with an estimated blood loss of 800 mL and no intraoperative hemodynamic instability. This is comparable to the blood loss reported across nearly 5,000 cesarean deliveries for various indications (792 mL) [17], despite the additional pelvic and femoral fixation performed during the same session. Achieving this outcome required close multidisciplinary planning among the obstetric, neonatology, anesthesia, and orthopedic teams, with the management strategy agreed upon before the patient entered the operating theater. The key considerations were that the fetus was near term, the pelvic injury carried an ongoing hemorrhagic risk, and a single combined procedure was preferable to two separate anesthetic events.

The concomitant open femoral fracture added complexity. Associated fractures occur in 48.5% of reported cases, and femoral shaft fractures, in particular, appear to be an adverse prognostic marker, with two of four mothers with such fractures in the pooled series not surviving [3]. The favorable outcome in this case highlights the value of coordinated multidisciplinary planning. However, this report describes a single case with short-term follow-up; long-term pelvic function, symphyseal healing, and the effects on future pregnancy remain unknown. Population data indicate altered subsequent obstetric management, including higher cesarean delivery rates, following pelvic fracture [18]. The absence of computed tomography also limits characterization of potential posterior pelvic ring involvement.

## Conclusions

Open-book pelvic fracture in pregnancy is rare and requires coordinated multidisciplinary management. Maternal stabilization remains the primary priority, and simultaneous cesarean section with pelvic stabilization can be safely performed in late gestation. This case highlights the importance of coordinated surgical planning in achieving favorable maternal and neonatal outcomes.
